# The relationship between disease activity with pan-immune-inflammatory value and systemic immune-inflammation index in rheumatoid arthritis

**DOI:** 10.1097/MD.0000000000037230

**Published:** 2024-03-01

**Authors:** Pinar Özge Başaran, Murat Dogan

**Affiliations:** a Physical Medicine and Rehabilitation Department, Erol Olcok Training and Research Hospital, Hitit University, Corum, Turkey; b Faculty of Medicine, Department of Internal Medicine, Hitit University, Corum, Turkey

**Keywords:** immune-inflammation value, inflammation index, Pan, Rheumatoid arthritis, Systemic immune

## Abstract

Rheumatoid arthritis (RA) is a chronic, systemic inflammatory disease. Immune system cells have an important role in RA. Our aim was to investigate the relationship between disease activity, systemic immune-inflammation index (SII), and pan-immune-inflammation value (PIV) levels in RA patients. We planned to investigate whether these 2 measurements have an advantage over each other. About 67 patients diagnosed with RA and 49 healthy controls included in this study. RA was diagnosed based on 2010 ACR classification criteria. In this cross-sectional study, peripheral blood tests, C-reactive protein (CRP), hemogram, and erythrocyte sedimentation rate levels were noted after the physical examination of all participants. PIV was calculated with the formula: (neutrophil count × platelet count × monocyte count) / lymphocyte count. SII was calculated as follows: (neutrophil count × monocytes count) / lymphocyte count. The disease activity score 28 (DAS28) were noted in patients with RA. CRP values of active RA group were significantly higher than remission RA and control groups (*P* < .001), control and remission RA groups were similar (*P* = .86). PIV and SII are significantly higher in active RA than remission RA and control (*P* < .001, *P* < .001) higher in remission RA than control (*P* < .001, *P* < .001). Receiver operating characteristic curve analysis in predicting remission compared to the control group, CRP was not significant, PIV and SII was significant and PIV has higher sensitivity and sensitivity, a PIV value of > 217.31 have sensitivity 75.0% and specificity 85.7%. CRP, PIV, and SII are statistically significant in predicting active RA compared to the remission RA and control group. Our findings show that PIV, and SII are easy, inexpensive and reliable markers predicting remission in RA patients. CRP was not significant compared to remission RA and control group, PIV and SII was significant and PIV has higher sensitivity and specificity than SII in the remission group in RA. Patients with high disease activity, PIV, SII, and CRP levels were effective in showing disease activity compared to RA remission group and healthy controls.

## 1. Introduction

Rheumatoid arthritis (RA) is a systemic, chronic, and inflammatory disease that mainly affects small joints, leads to structural, and functional impairments, and, as a result of all these, quality of life decreases.^[1]^ RA occurs in 0.5% to 1% of adults worldwide and. appears to be more common in women.^[2,3]^ RA occurs frequently in early adulthood. The diagnosis of RA is made using the 2010 ACR (American College of Rheumatology) diagnostic criteria.^[4]^ The first symptom usually manifested itself as joint involvement. Pain usually starts in the feet and hands at small joints, and stiffness in the morning that lasts several hours in these areas, which usually accompany the pain. Morning stiffness improves with activity, comes back with inactivity. The ethology is not fully known, In RA, inflammation begins in the synovial tissue and then damage develops to the subchondral tissue in the cartilage. As a result of this damage, the formation of the pannus in the tissue causes deformities and permanent deformities. Changes such as button marrow, swan neck, z-finger deformity also occur in the ligaments after impact. These deformities that occur in RA cause functional limitations and deterioration in quality of life.

Although there is no clarity at the RA pathogenesis, is thought to be mediated by the immune system. Many cytokines are involved in disease formation in RA. Tumor necrosis factor-alpha (TNF-α) plays an important role in RA pathogenesis.^[5]^ There is a strong response to this cytokine blockade in RA. Also, it contains high levels of TNF-α in synovial tissue in joints where inflammation is intense in the disease and is infiltrated by CD8 and CD4 T cells.^[6]^ In addition to TNF, other inflammatory cytokines, such as IL-1 (interleukin), IL-6, are also plays an important role at the pathogenesis of the disease.

Assessing the disease activity in RA is very important not only for preventing the major complications but also for early and effective treatment. Disease activity score (DAS28) is a commonly used score for determining disease activity and monitoring response to the treatment in RA.^[7]^

RA is a disease that affects the immune system and is accompanied by chronic inflammation. There is no specific laboratory test for RA diagnosis. The erythrocyte sedimentation rate (ESR) and the CRP (C-reactive protein) are commonly used in RA but have low sensitivity and specificity.^[8]^ Therefore, new immune-based prognostic scores like monocyte/lymphocyte ratio (MLR), neutrophil/lymphocyte (NLR) platelet/lymphocyte ratio (PLR) are studied in RA.^[9,10]^ Although each of these immune cells plays a role in inflammation, none of them is sufficient to detect inflammation alone. Because of these more comprehensive calculations involving these immune cells have been developed, like the pan-immune-inflammation value (PIV) and the systemic immune-inflammation index (SII).

SII is a marker calculated from a complete blood count that includes neutrophils, platelets, and lymphocytes and used to assess the level of inflammation. SII value increases with relatively high neutrophil and platelet counts and low lymphocyte counts and shows that, this is an indicator of a strong inflammatory response.^[11]^ SII has been evaluated in psoriatic arthritis, lupus, and RA, associated with disease activity level.^[12–14]^

PIV was first described by Fuca et al^[15]^ and used to evaluate the inflammation. PIV is calculated from 4 blood cell counts, including neutrophils, platelets, monocytes, and lymphocytes. In some rheumatological diseases such as RA, familial Mediterranean fever, and vasculitis, PIV has been studied.^[16–18]^

It is important to determine which blood parameter will be more effective in detecting disease activity and monitoring the treatment response in RA is important.

In our study, our aim was to investigate the relationship between RA disease activity and PIV and SII levels in RA. We planned to investigate whether these 2 measurements have an advantage over each other.

## 2. Material and methods

About 67 patients diagnosed with RA and 49 healthy controls included in this study. RA was diagnosed based on 2010 ACR classification criteria. This cross-sectional study was studied in patients diagnosed with RA in outpatient clinic at physical medicine and rehabilitation and whose treatment has not changed in the last 3 months were included. The control group consisted of patients without chronic or rheumatic diseases and admitted to the hospital for routine annual examination. In both the patient and control groups those who have malignancies, infections, immune deficiency, haematological diseases, other inflammatory diseases, or drug use that can affect blood parameters such as steroids or cytotoxic drugs were excluded. The study was carried out according to the principles of the Declaration of Helsinki, and written informed consent was obtained from all patients. Hitit University Faculty of Medicine Clinical Research Ethics Committee (2023-154) approved the study.

After the physical examinations of all patients, clinical and demographic and clinical features were recorded. The complete blood cell parameters neutrophils ((10^9^/L), lymphocytes (10^9^/L), monocytes (10^9^/L), platelets (10^9^/L), ESR (mm/h), and CRP (mg/L) levels were noted.

Systemic immune-inflammation index (SII) was calculated as follows: (neutrophil count × monocytes count) / lymphocyte count.^[11]^

Pan-immune-inflammation value (PIV) was calculated with the formula: (neutrophil count × platelet count × monocyte count) / lymphocyte count.^[15]^

DAS28 score was used for evaluating the disease activity level in RA patients. DAS28 is the most common used method in daily practice and is also recommended by the ACR and EULAR recommendations.^[7]^ In our study those with a DAS28 score of 2.6 and below were considered in remission, over 2.6 were considered in active disease.^[19]^

The sample size was calculated by the reference study, examining the total number of samples with the parameters, the effect size = 0.45, power (1-*β* error probability) = 0.95, α error probability = 0.05, and number of groups = 2 and total of 116 participants enrolled in the study (G-Power v3.1.9.7).^[20]^

### 2.1. Statistical analyses

IBM SPSS Statistics Standard Concurrent User V 29, statistical program was used for data analyses (IBM Corp., Armonk, NY). Percentage (%), number of units (n), mean ± standard deviation median, and inter-cartillary distance values are given as descriptive statistics. Intergroup comparisons for the age variable, one-way analysis of variance was used, and intergroup comparisons for other numerical variables were performed by Kruskal–Wallis test. Multiple comparisons in Kruskal–Wallis analysis were performed with Dunn–Bonferroni test. Gender distribution according to groups was analyzed by Pearson Chi-square. Receiver operating characteristic (ROC) Curve analysis was used for the performance of CRP, PIV, and SII variables in predicting diseases activity. The effect of CRP, PIV and SII variables on diseases was evaluated by multiple binary logistic regression analysis. Backward Wald was used as the elimination method. For statistically significancy, *P* < .05 was considered.^[21]^

## 3. Results

The study included 116 participants, 49 in the control group, 32 in the remission group, and 35 in the active RA group. The ages of the groups were not statistically different (*P* = 12). The number of male patients was 28 (57.1%) in the control group, 15 (46.9%) in the remission group, and 25 (71.4%) in the active RA group. Groups are similar in gender (*P* = .12; Table 1).

**Table 1:** Descriptive characteristics of all participants and comparison results of the groups.

	Groups			Test statistics	
	Control, *n* = 49	Remission RA, *n* = 32)	Active RA, *n* = 35	Test value	*P* value
Age	45.1 ± 9.7	40.7 ± 10.7	42.2 ± 9.2	2.120	0.125*
Gender *n* (%)					
Male	28 (57.1)	15 (46.9)	25 (71.4)	4.231	0.121†
Female	21 (42.9)	17 (53.1)	10 (28.6)		
Sedimentation rate	7.00 (7.50)	8.00 (8.50)	11.00 (17.00)	3.783	0.151‡
Hemoglobin	15.20 (2.50)^a^	13.75 (2.88)^b^	13.90 (2.50)^a^^,^^b^	6.886	**0.032** ‡
Neutrophil	3.74 (1.95)^a^	4.25 (1.36)^a^	5.76 (2.08)^b^	39.109	**<0.001** ‡
Monocyte	0.40 (0.20)^a^	0.57 (0.05)^b^	0.57 (0.11)^b^	36.451	**<0.001** ‡
Lymphocyte	2.52 (0.54)^a^	2.33 (0.72)^a^	1.86 (0.76)^b^	27.124	**<0.001** ‡
Neutrophil percent	52.90 (10.77)^a^	56.70 (9.43)^b^	57.40 (11.50)^b^	8.037	**0.018** ‡
Lymphocyte percent	37.60 (12.91)^a^	32.85 (8.53)^a^^,^^b^	30.60 (7.30)^b^	7.000	**0.030** ‡
Platelet	228.0 (78.5)	258.0 (70.2)	252.0 (74.0)	2.873	0.238‡
MPV	10.30 (1.15)	10.10 (1.23)	10.10 (1.14)	2.247	0.325‡
RDW	13.10 (1.40)^a^	13.80 (1.35)^b^	14.20 (1.50)^b^	15.946	**<0.001** ‡
CRP	3.11 (0.98)^a^	3.16 (0.91)^a^	12.80 (14.00)^b^	34.463	**<0.001** ‡
PIV	125.7 (119.3)^a^	240.2 (105.6)^b^	455.99 (362.0)^c^	62.796	**<0.001** ‡
SII	0.58 (0.33)^a^	0.95 (0.56)^b^	1.74 (0.88)^c^	65.755	**<0.001** ‡

*P* values in bold indicate statistical significant.

*n*: number of patients, %:percentage, the numerical data are given as mean ± standard deviation or median (interquartile range)

MPV = mean platellt volüme, RDW = red cell distrubition width, CRP = C-reactive protein, PIV = pan-immüne-inflammation value, SII = systemic immune-inflammation index

* : One-way variance, †: Pearson chi-square test, ‡: Kruskal-Wallis H test, ^a^, ^b^, and ^c^ superscripts indicate differences between groups in each row. There is no statistical difference between groups with the same superscript.

Neutrophil values of the active RA group are statistically higher than those of the remission RA and control group (*P* < .001). The neutrophil values are similar in remission RA and control groups. The monocyte values of remission RA, and active RA groups are statistically higher than the control group (*P* < .001). Monocyte values of remission and active RA groups are not statistically different. The lymphocyte values of active RA group are statistically lower than those of the other 2 groups (*P* < .001). Lymphocyte values of the control and remission groups are similar.

The CRP, PIV, and SII values of the groups are statistically different. CRP values of active RA group are statistically higher than control and remission groups (*P* < .001). The CRP values of the control and remission groups are similar (*P* = .86).

PIV values of active RA group are statistically higher than remission and control groups (*P* < .001). PIV values of remission group are statistically higher than control group (*P* < .001).

SII values of active RA group are statistically higher than remission and control groups the groups (*P* < .001). SII values of the remission group are statistically higher than control group (*P* < .001).

**Figure 1.**
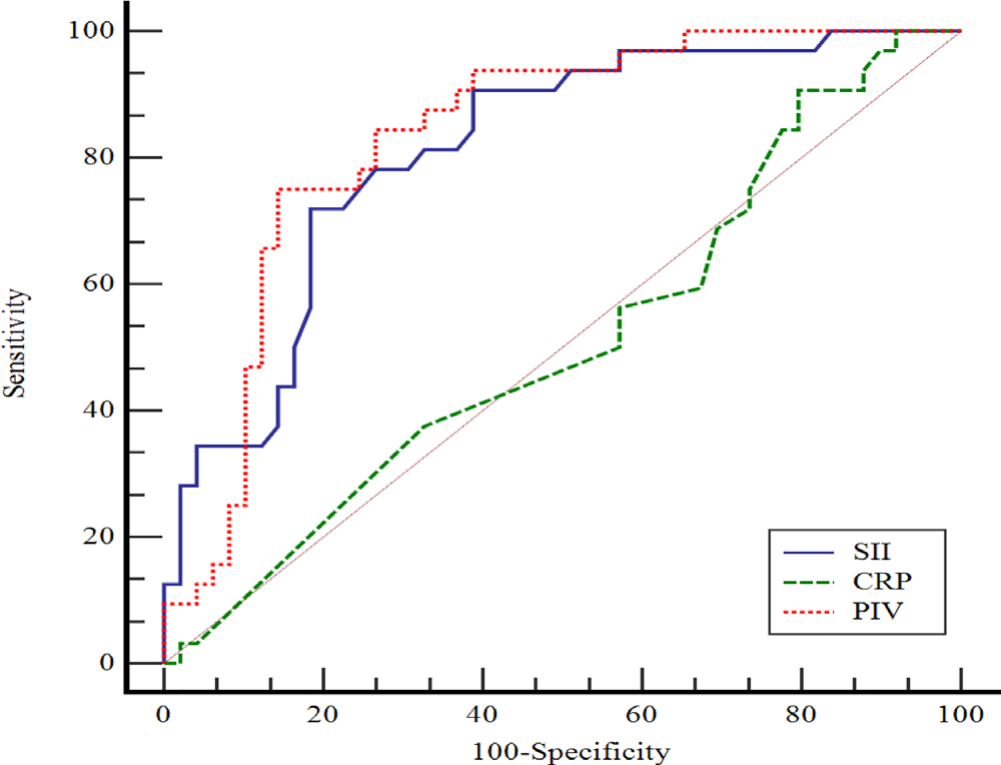
ROC curve for remission RA compared to control group.

In Figure 1, the performance of CRP, PIV, and SII values in predicting remission compared to the control group was assessed by ROC curve analysis. The area under the curve (AUC) value for CRP is not statistically significant. AUC values for PIV and SII are statistically significant. When the PIV value was > 217.31, sensitivity was 75.0% and specificity was 85.7%. When the SII value was > 0.82, sensitivity was 71.8% and specificity was 81.6% (Table 2).

**Table 2:** Evaluation of the performance of CRP, PIV and SII for remission group (compared to control group) by ROC curve analysis.

	AUC (95.0% CI)	*p*	Cutoff	Sensitivity (95.0% CI)	Specificity (95.0% CI)
CRP	0.511 (0.398–0.624)	0.865	≤4.7	90.6 (75.0–98.0)	20.4 (10.2–34.3)
PIV	0.834 (0.735–0.908)	<0.001	>217.31	75.0 (56.6–88.5)	85.7 (72.8–94.1)
SII	0.811 (0.709–0.889)	<0.001	>0.82	71.8 (53.3–86.3)	81.6 (68.0–91.2)

AUC = area under the curve, CI = confidence interval

**Figure 2.**
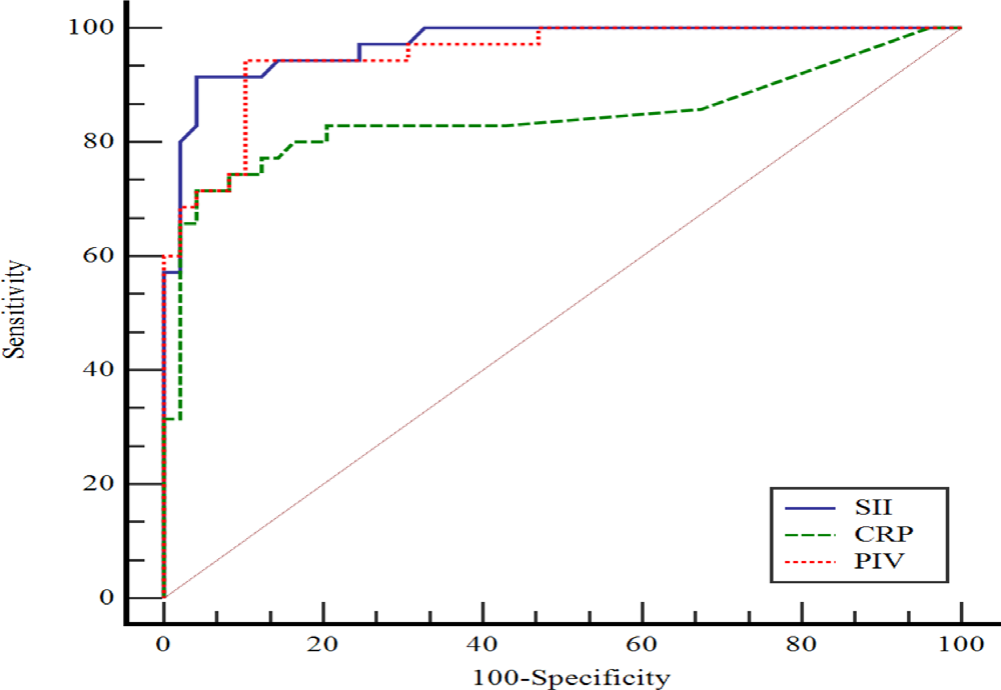
ROC curve for active RA compared to control group.

In Figure 2, the performance of CRP, PIV, and SII values in predicting active RA compared to the control group was assessed by ROC curve analysis. AUC values for CRP, PIV, and SII are statistically significant. When CRP value was > 8, sensitivity was 71.4% and specificity 95.9%; when PIV value was > 243.97, sensitivity was 97.2% and specificity was 89.8%. When SII value was > 1.08, specificity was 95.9%, sensitivity was 91.4% (Table 3).

**Table 3:** Evaluation of the performance of CRP, PIV and SII for active RA Group (compared to control group) by ROC curve analysis.

	AUC (95.0% CI)	*p*	Cutoff	Sensitivity (95.0% CI)	Specificity (95.0% CI)
CRP	0.842 (0.746–0.913)	<0.001	>8	71.4 (53.7–85.4)	95.9 (86.0–99.5)
PIV	0.952 (0.882–0.987)	<0.001	>243.97	97.2 (80.8–99.3)	89.8 (77.8–96.6)
SII	0.971 (0.909–0.995)	<0.001	>1.08	91.4 (76.9–98.2)	95.9 (86.0–99.5)

AUC = area under the curve, CI = confidence interval

**Figure 3.**
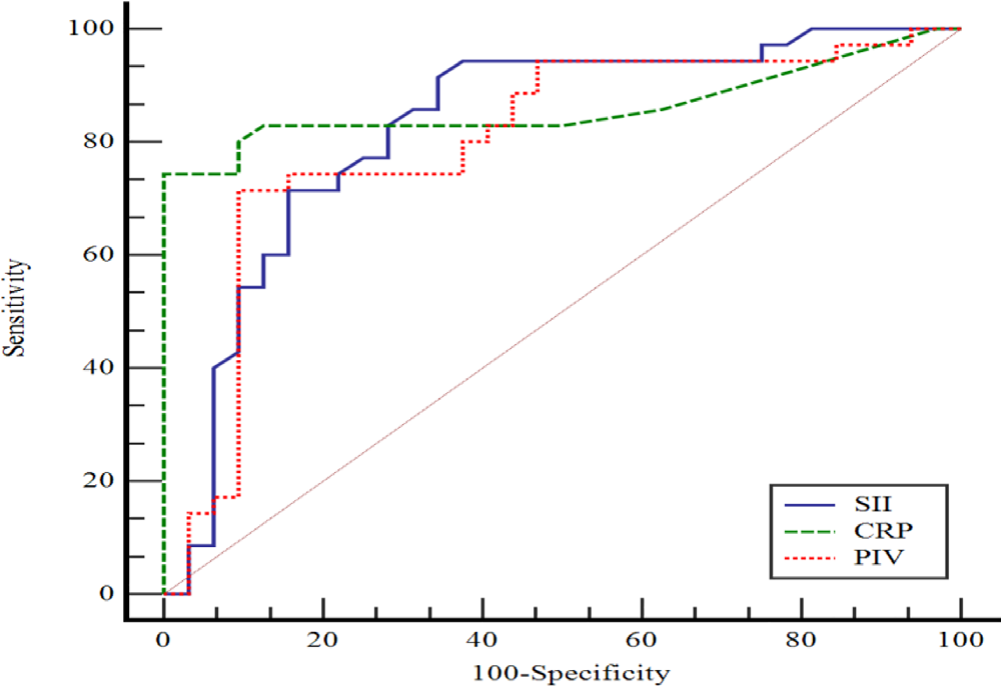
ROC curve for active RA compared to remission group.

In Figure 3, the performance of CRP, PIV, and SII values in predicting active RA compared to the remission group was assessed by ROC curve analysis. AUC values for CRP, PIV, and SII are statistically significant. When CRP value was > 6.7, sensitivity was 74.2% and specificity was 100.0%; when PIV value was > 352.4, sensitivity was 71.4% and specificity was 90.6%. When SII value was > 1.02, specificity was 65.6%, sensitivity was 91.4% (Table 4).

**Table 4:** Evaluation of the performance of CRP, PIV and SII for active RA group (compared to remission group) by ROC curve analysis.

	AUC (95.0% CI)	*p*	Cutoff	Sensitivity (95.0% CI)	Specificity (95.0% CI)
CRP	0.862 (0.755–0.934)	<0.001	>6.7	74.2 (56.7–87.5)	100.0 (89.1–100.0)
PIV	0.803 (0.687–0.890)	<0.001	>352.4	71.4 (53.7–85.4)	90.6 (75.0–98.0)
SII	0.828 (0.716–0.909)	<0.001	>1.02	91.4 (76.9–98.2)	65.6 (46.8–81.4)

AUC = Area under the curve, CI = Confidence interval

In the analyses in Table 5; PIV, CRP, and SII variables were included in all 3 models. According to the analysis between remission and control group in Model 1, PIV was ultimately found to be effective. As the PIV value increases, the risk of remission increases 1.011-times. According to the analysis between active RA and control group in Model 2, CRP and SII variables were found to be effective. As CRP increases, the risk of active RA increases 1.244-times, and as SII increases, the risk of active RA increases 67.128-times. According to the analysis between active RA and remission groups in Model 3, CRP was found to be statistically effective. As CRP increases, the risk of active RA increases 1.811-times.

**Table 5:** Evaluation of the effects of CRP, PIV, and SII on remission and active RA groups with multiple binary logistic regression analyses.

	Regression coefficients*						
	*β*	S.E.	Wald statistics	*p*	Exp(*β*)	95% C.I. for exp(*β*)	
						Lower	Upper
Model 1: remission vs control							
Constant	−2.818	0.683	17.011	<0.001^*^	0.060		
PIV	0.011	0.003	13.196	<0.001^*^	1.011	1.005	1.017
Model 2: active RA vs control							
Constant	−6.543	1.379	22.525	<0.001^*^	0.001		
CRP	0.219	0.102	4.568	0.033^*^	1.244	1.018	1.520
SII	4.207	1.092	14.848	<0.001^*^	67.128	7.901	570.356
Model 3: active RA vs remission							
Constant	−3.373	0.885	14.515	<0.001^*^	0.034		
CRP	0.594	0.189	9.895	0.002^*^	1.811	1.251	2.623

Elimination method: Backward Wald, *P* values in bold indicate statistical significance (*P < 0.05 was statistical significant).

## 4. Discussion

Different blood parameters have been studied in RA such as NLR, PLR, PIV, and SII. To our knowledge this is the first study to compare PIV, and SII values in patients with RA. As a result of our study, CRP was not effective in showing disease activity in remission in RA compared to the healthy controls, while PIV, and SII values were found to be effective. PIV has higher sensitivity and specificity than SII in the remission group in RA. In RA patients with high disease activity levels, CRP, PIV, and SII are effective in showing disease activity compared to RA remission group, and healthy controls.

In RA, severe deformities develop in the hands and feet, especially in small joints. These deformities often develop in patients with active disease by intense inflammation. Functional losses develop as a result of deformities and the patient’s quality of life decreases. Therefore, it is important to follow the disease activity in RA. Additionally, the response to the treatment used in RA is determined according to disease activity and the drug selection is made accordingly.

CRP and ESH are noninvasive biomarkers for the purpose of evaluating disease activation in patients with RA and have been used for many years.^[8]^ CRP used as an acute phase protein; its measurement is relatively cheap, common, and easy; however, elevated serum CRP levels it may also be affected by other inflammatory processes. ESH response later and slower to disease activity changes, it is less widely used than the CRP test due to its responsiveness. Because of all this, researchers search for fast, cheap and easy markers to evaluate disease activation. In RA, a constant inflammatory stimulus leads to impaired function of neutrophils, platelets, and lymphocytes. B and T lymphocytes are important components of the immune system. In RA, hyperactivity of B lymphocytes and dysfunction of T lymphocytes, results with immune deflection.^[22]^ Overstimulation of T lymphocytes leads to their depletion. B lymphocytes and related autoantibody production increase.^[23]^ In recent years, some systemic inflammatory markers obtained from more complex complete blood count markers have been developed to detect these changes, such as NLR and PLR. SII and PIV are also among these inflammatory markers. SII includes neutrophils, platelets, and lymphocytes. Unlike SII, PIV also contains platelets, which are part of natural immunity.

The predictive value of PIV, and SII in indicating disease activity has been investigated in the studies.^[16–18]^ Also PIV and SII investigated for investigated the predictability of malignancy prognosis.^[24–26]^

Satiş^[27]^ evaluated a relationship with SII and disease activity levels in patients with RA, but CRP and other inflammatory parameters were not considered in the study. Likewise our study; SII levels were higher in active RA patients have higher SII levels than patients with remission RA.

SII and PIV are not only related to disease activity but can also be used to monitor response to the treatment. In a previous study, CRP, ESR, and SII were found to be successful in monitoring response to treatment in patients with RA and the predictive value of SII was found to be the highest compared to CRP, and ESR.^[28]^ Compared to the previous study, the sensitivity and specificity of SII were found to be lower than in our study.^[28]^ They performed statistical analysis only in the patients with RA on anti-TNF treatment. This study did not include healthy controls. Patients with and without anti-TNF treatment were compared and since it was a retrospective study, only patients registered in the system were examined. We examined the relationship between SII, PIV, and CRP in active, remission, and healthy controls and when SII increases the risk of active RA increases more compared to healthy controls. Liu et al^[14]^ found similar results in RA patients and healthy population, but only examined the patients in terms of SII values and did not evaluate them in terms of other inflammation parameters and disease activity.

Fuca et al^[15]^ demonstrated that PIV score outperformed other immunoinflammatory biomarkers such as neutrophil-lymphocyte ratio, monocyte count, platelet count, and SII in logistic regression analysis in colorectal cancer patients. In a meta-analyses, it has been shown that PIV may be a prognostic marker in cancer patients.^[29]^

When we searched the literature, we could only find 1 study that evaluated the relationship between PIV and RA. In this study, PIV was found to be effective in differentiating active RA from remission and control group likewise our study.^[17]^ As shown in studies, CRP, PIV, and SII 3 inflammatory blood parameters increase during the period of high activation in RA. It is hard to determine the parameter to be used in the remission phase, and in monitoring the response to treatment. In our study CRP is not statistically significant comparing remission RA with control group, but PIV and SII values are significant and also PIV was found to be effective with remission.

The relationship between PIV and SII has previously been studied in patient with colorectal cancer, and both parameters were found to be elevated compared to healthy controls.^[30]^ In a study conducted on sarcoidosis, and seen that PIV and SII values were similar in patients with sarcoidosis and controls.^[31]^ PIV and SII were higher in patients with malignant melanoma receiving immunotherapy than the healthy controls, and PIV showed some power to differentiate patients with metastases from patients with stage 1–2 malignant melanoma.^[26]^ In another study conducted in patients with idiopathic membranous nephropathy, PIV and SII were successful in differentiating low and moderate nephropathy.^[32]^

Our work has some advantages. First, it is the first study to examine the relationship between PIV, SII, and CRP. In our study, both patients with active RA, RA in remission and healthy controls were evaluated and the sensitivity and specificity of inflammation parameters and their superiority over each other were analyzed in each group.

However, our study is a single-center study and our sample size is small. The medications used by the patients were ignored. Response to treatments was not evaluated and there was no follow-up period.

Studies in larger groups of patients are also required in which the drugs used by patients are evaluated.

## 5. Conclusion

PIV and SII are easy, inexpensive, and reliable markers predicting remission in RA patients. CRP was not significant compared to remission RA and control group, PIV and SII was significant and PIV has higher sensitivity and specificity than SII in the remission group in RA. In RA patients with high disease activity, CRP, PIV, and SII are effective in showing disease activity compared to healthy controls and RA remission group.

## Author contributions

**Conceptualization:** Pinar Ozge Basaran, Murat Dogan.

**Data curation:** Pinar Ozge Basaran, Murat Dogan.

**Formal analysis:** Pinar Ozge Basaran, Murat Dogan.

**Funding acquisition:** Pinar Ozge Basaran, Murat Dogan.

**Investigation:** Pinar Ozge Basaran, Murat Dogan.

**Methodology:** Pinar Ozge Basaran, Murat Dogan.

**Project administration:** Pinar Ozge Basaran, Murat Dogan.

**Resources:** Pinar Ozge Basaran, Murat Dogan.

**Software:** Pinar Ozge Basaran, Murat Dogan.

**Supervision:** Pinar Ozge Basaran, Murat Dogan.

**Validation:** Pinar Ozge Basaran, Murat Dogan.

**Visualization:** Pinar Ozge Basaran, Murat Dogan.

**Writing—original draft:** Pinar Ozge Basaran, Murat Dogan.

**Writing—review & editing:** Pinar Ozge Basaran, Murat Dogan.
